# The Role of Epidermal Growth Factor Receptor Family of Receptor Tyrosine Kinases in Mediating Diabetes-Induced Cardiovascular Complications

**DOI:** 10.3389/fphar.2021.701390

**Published:** 2021-08-02

**Authors:** Bara A. Shraim, Moaz O. Moursi, Ibrahim F. Benter, Abdella M. Habib, Saghir Akhtar

**Affiliations:** ^1^ College of Medicine, QU Health, Qatar University, Doha, Qatar; ^2^ Biomedical and Pharmaceutical Research Unit, QU Health, Qatar University, Doha, Qatar; ^3^ Faculty of Medicine, Eastern Mediterranean University, Famagusta, North Cyprus

**Keywords:** epidermal growth factor receptor, ErbB2, ErbB3, ErbB4, diabetes, heart, cardiac dysfunction, vascular dysfunction

## Abstract

Diabetes mellitus is a major debilitating disease whose global incidence is progressively increasing with currently over 463 million adult sufferers and this figure will likely reach over 700 million by the year 2045. It is the complications of diabetes such as cardiovascular, renal, neuronal and ocular dysfunction that lead to increased patient morbidity and mortality. Of these, cardiovascular complications that can result in stroke and cardiomyopathies are 2- to 5-fold more likely in diabetes but the underlying mechanisms involved in their development are not fully understood. Emerging research suggests that members of the Epidermal Growth Factor Receptor (EGFR/ErbB/HER) family of tyrosine kinases can have a dual role in that they are beneficially required for normal development and physiological functioning of the cardiovascular system (CVS) as well as in salvage pathways following acute cardiac ischemia/reperfusion injury but their chronic dysregulation may also be intricately involved in mediating diabetes-induced cardiovascular pathologies. Here we review the evidence for EGFR/ErbB/HER receptors in mediating these dual roles in the CVS and also discuss their potential interplay with the Renin-Angiotensin-Aldosterone System heptapeptide, Angiotensin-(1-7), as well the arachidonic acid metabolite, 20-HETE (20-hydroxy-5, 8, 11, 14-eicosatetraenoic acid). A greater understanding of the multi-faceted roles of EGFR/ErbB/HER family of tyrosine kinases and their interplay with other key modulators of cardiovascular function could facilitate the development of novel therapeutic strategies for treating diabetes-induced cardiovascular complications.

## Introduction

Diabetes mellites (DM) is a set of metabolic disorders arising from defective insulin secretion and/or action in which hyperglycemia (HG) is a common feature. Type 1 diabetes mellitus (T1DM) results from the autoimmune-mediated destruction of pancreatic β cells. The infiltration of T lymphocytes, release of cytokines, and generation of reactive oxygen species (ROS) ultimately results in the dysfunction and apoptosis of pancreatic β cells (Morgan et al., 2014; Crevecoeur et al., 2015; Tan et al., 2019; Toi et al., 2020). In contrast, type 2 diabetes mellitus (T2DM) is characterized by target tissue resistance to the metabolic actions of insulin as well as pancreatic β cell dysfunction. Insulin resistance is strongly associated with visceral obesity and the resulting chronic low-grade inflammation as well as increased ectopic fat deposition (Saltiel, 2021; Tsatsoulis, et al., 2013).

The International Diabetes Federation declared that approximately 463 million adults are currently living with diabetes mellitus and this figure will likely reach over 700 million by the year 2045. Half of the sufferers are unaware of their condition and hence, prone to serious hyperglycemia/diabetes-related complications (IDF Diabetes Atlas, 2019). DM results in secondary pathophysiologic alterations in several organ systems that impose an immense burden on both the individual with diabetes and the public health system. Cardiovascular (CV) complications constitute the leading cause of morbidity and mortality in individuals with DM (Forbes and Cooper, 2013; Babel and Dandekar, 2021). These include microangiopathy, abnormal vascular reactivity, atherosclerosis, hypertension, cardiomyopathy, ischemic heart disease, and myocardial infarction (Akhtar and Benter, 2013; Babel and Dandekar, 2021). Moreover, the specific underlying mechanisms leading to the development of these CV complications are not fully understood and likely involve multiple intracellular signaling networks that can be activated by hyperglycemia (HG) and/or diabetes.

Epidermal growth factor receptor (EGFR/ErbB1/HER1), one of the most versatile signaling units in biology, plays a key role in regulating many cellular functions including growth, proliferation, motility, and survival (Kumagai et al., 2021; Sharifi et al., 2021). Perturbations in EGFR expression and signalling are implicated in several pathophysiological conditions including cancer and diabetes (Matroughi, 2010; Akhtar and Benter, 2013; Xu et al., 2017; Akhtar et al., 2018; Talukdar et al., 2020; Kumagai et al., 2021; Sharifi et al., 2021). Emerging research suggests that members of the Epidermal Growth Factor Receptor (EGFR/ErbB/HER) family of tyrosine kinases can have a dual role in that they are necessary (acting as “good guys”) for the normal development and physiological functioning of the cardiovascular system (CVS) but their dysregulation may also be intricately involved in mediating cardiovascular pathologies (i.e., acting as “bad guys”) (Matrougui, 2010; Akhtar and Benter, 2013; Schreier et al., 2014). In this article, we review the evidence for EGFR/ErbB/HER receptors in mediating these dual (“good guy” verses “bad guy”) roles in the heart and then in the vascular system followed by a discussion of their potential interplay with the Renin-Angiotensin-Aldosterone System heptapeptide, Angiotensin-(1–7), as well the arachidonic acid metabolite, 20-HETE-key players in the regulation of cardiovascular function.

## Epidermal Growth Factor Receptor Family of Receptor Tyrosine Kinases: An Overview

EGFR (a/k/a ErbB1 or HER1) is a 180 kDa transmembrane glycoprotein made up of 1,186 amino acids that belongs to the broader family of receptor tyrosine kinases (RTKs) (Rayego-Mateos et al., 2018; Kumagai et al., 2021). It is made up of four conserved domains: an extracellular ligand-binding domain, a transmembrane domain, a cytoplasmic tyrosine kinase-containing domain, and a regulatory domain (Zheng et al., 2014 ). Moreover, there are three other homologous members that comprise the EGFR/ErbB/HER family of RTKs: ErbB2 (EGFR2/HER2/Neu), ErbB3 (EGFR3/HER3), and ErbB4 (EGFR4/HER4). EGFR members, with the exception of ErbB2, are activated by numerous growth factors or ligands such as epidermal growth factor (EGF), betacellulin, neuregulin-1 (NRG-1) and Heparin-binding EGF-like growth factor (HB-EGF). Upon ligand interaction with its cognate receptor, either homodimerization of the receptor or heterodimerization with other members of the EGFR family occurs. ErbB2 lacks a known ligand and depends on heterodimerization with other receptors of the EGFR family for activation. ErbB3 lacks a functional kinase domain and therefore also relies on heterodimerization with other EGFR/ErbB receptors for activity. Thus, EGFR and ErbB4 receptors are the only fully functioning homodimers (Kumagai et al., 2021; Sharifi et al., 2021). Next, phosphorylation of precisely defined tyrosine residues within the cytoplasmic kinase portion of the receptor dimer occurs, eventually leading to the activation of multiple downstream signaling cascades. Such cascades include p38 Mitogen-activated protein kinase (MAPKs), Extracellular-signal regulated kinase 1/2 (ERK1/2), Phosphatidylinositol 3-kinases (PI3Ks)/Protein kinase B (AKT), phospholipase C-gamma 1 (PLCγ1), and Janus kinase (JAK) (/Signal transducers and activation of transcription (STAT) pathways (Rayego-Mateos et al., 2018). Consequently, several cellular processes, such as angiogenesis, proliferation, differentiation, motility, survival, and apoptosis can take place (Akhtar and Benter, 2013; Kumagai et al., 2021; Sharifi et al., 2021; Zheng et al., 2014).

EGFR/ErbBs can also be activated via ligand-independent pathways through a process of “transactivation” such as by peptides of the Renin-Angiotensin-Aldosterone system (RAAS) and other G-protein coupled receptors (GPCRs) (Forrester et al., 2016). For example, EGFR transactivation can occur via Angiotensin II (Ang II) Norepinephrine (NE), (leptin, thrombin, and endothelin by mechanisms involving non-ligand associated Src family kinases, and/or mediated via matrix metalloproteases (MMP), and/or a disintegrin and metalloprotease (ADAM)-dependent shedding of cell surface bound EGF-like ligands (Matrougui, 2010; Akhtar et al., 2012a; Beltowski and Jazmroz-Wisniewska, 2014; Chen et al., 2015; Forrester et al., 2016; Reichelt et al., 2017). It should be noted that all ligands of the EGFR/ErbB family of receptors exist as membrane-anchored precursors that are released by enzymatic-cleavage by sheddases such as MMPs and ADAMs (Rayego-Mateos et al., 2018). EGFR/ErbB receptors are expressed widely in epithelial, neuronal and mesenchymal tissues (Yano et al., 2003; Chen et al., 2016). Hence, EGFR/ErbBs are key regulators of many cellular homeostatic functions and their dysregulation is associated with several pathological functions including diabetes-induced cardiovascular dysfunction.

## Diabetes-Induced Cardiac Dysfunction: The Dual Role of Epidermal Growth Factor Receptor Family of Receptor Tyrosine Kinases and Their Ligands in the Heart

Cardiac complications including diabetic cardiomyopathy and heart failure are 2- to 5-fold more likely in patients with diabetes (for a review see Kenny and Abel, 2019). This increased risk is dependent on blood glucose levels as higher HbA1c levels (indicating worsening hyperglycemia) are associated with increased likelihood of heart failure in patients with diabetes (Erquo et al., 2013). Even short-term episodes of hyperglycemia or “glycemic variability” such as those observed postprandially are thought to increase the risk of developing cardiovascular complications associated with diabetes (Ceriello, 2005; Brownlee and Hirsch, 2006). Interestingly, in long-term studies it has been shown that subsequent therapy-induced correction of hyperglycemia does not normalise the risk of developing cardiovascular complications back to baseline (Kenny and Abel, 2019) implying that even transient excursions into hyperglycemia may induce long-term adverse molecular or signaling changes in the cardiovascular system that can lead to cardiovascular complications. This phenomenon has been described as “glycemic” or “metabolic” memory (Chalmers and Cooper, 2008; Holman et al., 2008); though recently this concept has been challenged (Miller and Orchard, 2020). Diabetes-induced cardiac complications may arise from a direct effect of hyperglycemia-induced molecular changes in the cardiac muscle as observed in cardiac myopathy (hypertrophy and fibrosis) or in the microvasculature of the heart that manifest as atherosclerosis or coronary artery disease (Falcao-Pires and Leite-Moreira, 2012; Kenny and Abel, 2019; Babel and Dandekar, 2021). Patients with diabetes who develop cardiomyopathy initially exhibit a hidden subclinical phase of myocardial remodeling that leads to compromised diastolic function, followed by systolic dysfunction that can progress to eventual heart failure (Falcao-Pires and Leite-Moreira, 2012) The exact underlying mechanisms for the development of cardiac pathologies in diabetes are not fully understood but likely involve a wide array of cell signaling networks. These include generation of reactive oxygen species (ROS)/oxidative stress, endoplasmic reticulum (ER) stress, mitochondrial dysfunction, accumulation of advanced glycated end-products (see also section on vascular dysfunction), and hyperglycemia-induced alterations in signaling by growth factors, peptides of RAAS as well as other GPCRs that are known also to transactivate EGFR (for recent reviews see Babel and Dandekar, 2021; Kenny and Abel, 2019, Forrester et al., 2016). Emerging evidence suggests that not only do EGFR/ErbB/HER family of TK receptors play a key role in the development of diabetes-induced cardiac dysfunction but they may also represent a convergent point or “hub” and a “relay” for the multiple other signaling molecules or inputs leading to cardiac dysfunction. In fact, the current understanding is that ErbBs may play a dual role, whereby they can elicit beneficial as well as detrimental signaling in the heart. In the proceeding section, we will discuss studies highlighting the dual role of EGFR/ErbB/HER receptor signaling in the normal (physiological) and the diabetic (pathological) heart.

### The Beneficial Role of Epidermal Growth Factor Receptors in the Normal and Diabetic Heart

EGFR/ErbB/HER receptors are vitally important in the physiological development of the heart irrespective of whether it is normal or diabetic. Indeed, the beneficial (good guy) role of ErbB receptors starts even before birth. These receptor tyrosine kinases (RTKs) are essential for normal cardiac morphogenesis during embryogenesis, and also in maintaining proper physiology of the adult heart (Sanchez-Soria and Camenisch, 2010; Fuller et al., 2008) see also Table 1. Observations in engineered knockout (KO) or mutant mice of ErbB1, ErbB2, ErbB3, ErbB4 highlighted the lethal phenotypes and heart defects that can arise as a result of a deficiency in the number or signaling of these receptors (for review see also Sanchez-Soria and Camenisch, 2010). ErbB1 KO and mutant mice displayed semilunar valve defects (Sibilia and Wagner, 1995; Chen et al., 2000) whilst disrupted endocardial cushion/heart valve mesenchyme formation was noted in ErbB3 KO mice that ultimately leads to death of embryos within 2 weeks (Erickson et al., 1997; Camenisch et al., 2002). ErbB2 and ErbB4 KO mice lack ventricular trabeculation-a key process in the maturation of ventricles and necessary for physical contractility and normal blood flow (Gassmann et al., 1995; Lee et al., 1995; Chan et al., 2002; Negro et al., 2004).

**TABLE 1:** A summary of selected studies highlighting the **beneficial** role of EGFR signaling in the heart.

Role of EGFR	Study model	Intervention	Results	Refs.
**ErbB1 expression protects from myocardial I/R injury**	Myocyte-specific Hif2a or ErbB1 knockout mice		• RNA-binding protein 4 suppression attenuated hypoxia-inducible factor 2A-dependent induction of ErbB1	(Lee et al., 2020)
			• ErbB1 myosin Cre + mice suffered larger infarctions and could not be saved by amphiregulin	
**EGF protects against myocardial I/R injury**	C57BL/6 (B6) mice	EGF	• EGF inhibits ROS and H_2_O_2_ induced cell death and by Nrf2 activation	Ma and Jin, (2019)
			• EGF limited cardiac I/R injury and apoptosis *in vivo*	
**NRG-1 receptor ErbB3 limits apoptosis and improves cell survival**	Wistar rats	I/R injury and protective post-conditioning procedure	• ErbB3 expression increased after I/R injury (with and without post-conditioning)	Morano et al. (2017)
			• ErbB3 expression improved cell survival and reduced mitochondrial dysfunction and apoptosis	
**NRG-1β induces proliferation, survival and paracrine signaling**	Primary human cardiac ventricular fibroblasts	NRG-1β	• NRG-1β improved proliferation and survival of human cardiac fibroblasts by inducing ErbB3-dependent activation of ErbB2	Kirabo et al. (2017)
**NRG-1 inhibits ER stress and protects against myocardial I/R injury**	Sprague-dawley and wistar rats	NRG-1 ligand of cardiomyocyte ErbB receptors	• NRG-1 reduced cardiomyocyte ER stress, hypoxia-reoxygenation induced apoptosis and myocardial infarct size induced by I/R injury	Fang et al. (2017)
**ErbB activation alleviates doxorubicin induced cardiac toxicity**	Stem cell derived human cardiac myocytes	Trastuzumab and lapatinib	• ErbB activation with NRG protected against doxorubicin-induced cardiac myocyte injury, while inhibition with trastuzumab exacerbated it	Eldridge et al. (2014)
**EGFR/ErbB2 improves T1D hearts recovery from I/R injury**	Wistar rat	AG825 or AG1478	• Chronic AG1478 or AG825 treatment decreased cardiac recovery in normal and diabetic rats	Akhtar et al. (2012b)
		EGF and/or losartan	• Acute EGF treatment pre or post ischemia improved cardiac recovery and opposed ischemic changes by EGFR/ErbB2 activation in T1D hearts	
**EGFR protects myocytes in reperfused hearts**	C57/Bl6 mice	AG1478, GM6001, or CRM197	• EGFR inhibition limited CCPA-mediated functional protection in reperfused hearts	Williams-Pritchard et al. (2011)
**EGFR maintains normal cardiac function and left ventricular thickness**	C57BL/6J (B6) mice	EGFR small molecule TKIs, irreversible EKB-569 and reversible AG1478	• EGFR inhibition resulted in left ventricular thickness and cardiac function changes via increasing fibrosis and altering left ventricular apoptotic gene expression	Barrick et al. (2008)
**ErbB4 plays a major role in normal cardiac conduction and ventricular trabeculation**	ErbB4 cardiac-knockout mice		• ErbB4 deletion resulted in severe dilated cardiomyopathy, abnormal conduction, impaired ventricular trabeculation and premature death	Garcia-Rivello et al. (2005)
**EGFR/ErbB1 activation protects against stress-induced cardiac injury**	Adult Swiss-CD1 male mice	EGF, AG1478	• EGF led to lower increase in total LDH, LDH-1, and creatinine kinase activity, and protected against stress-induced cardiac injury (these effects were abolished by simultaneous AG1478 administration)	Pareja et al. (2003)
**ErbB2 prevents dilated cardiomyopathy**	C57BL/6J mice		• Ventricular-restricted ErbB2 deletion resulted in dilated cardiomyopathy with impaired left ventricular contractility and increased susceptibility of cardiomyocytes to anthracycline toxicity	Crone et al. (2002)

I/R, ischemia/reperfusion; NRG, neuregulin; ER, endoplasmic reticulum; T1D, type-1 diabetes; CCPA, 2-chloro-N(6)-cyclopentyladenosine; CK, creatinine kinase.

ErbB2 is also important for neural development and myelination of nerve fibres (Lee et al., 1995; Garratt et al., 2000). Not only does ErbB2 signaling participate in the development of the ventricular conduction system (Negro et al., 2004) but it is also required for cardiac atrial electrical activity during development of the atrial conduction system that if disrupted leads to premature death at mid-gestation (Tenin et al., 2014). Thus, activation of ErbB signaling has been proposed as a therapeutic strategy to reduce the mortality rate of congenital heart diseases and in the prevention of cardiac damage in adults (Sanchez-Soria and Camenisch, 2010). Collectively, these studies suggest that all four EGFR/ErbB/HER receptors are necessary for the proper physiological development of the heart-the first organ to form during embryogenesis. Furthermore, all EGFR/ErbB/HER receptors and several of their ligands are now also thought to be expressed in the adult heart (either in cardiac myocytes, fibroblasts, endothelial or vascular smooth muscle cells) and are required for normal physiological functioning of the post-natal heart (Camprecios et al., 2011; Kirabo et al., 2017; Sanchez-Soria and Camenisch, 2010). Of the EGFR/ErbB receptors, ErbB2 and ErbB4 appear to be the most abundant and of the ligands, EGF, HB-EGF and NRG-1 are thought to be most important in regulating heart function (Reichelt et al., 2017).

The EGFR/ErbB1 receptor and some of its ligands, especially EGF and HB-EGF, are expressed in the adult heart and thus, both ligand-activated signaling and ligand-independent transactivation of EGFR likely contribute to specific protective responses in the adult heart (for review see Reichelt et al., 2017). EGFR/ErbB1 receptor appears to be important in cardiomyocyte survival and contractile function with its cardiac-specific deletion in the adult heart resulting in cardiac dysfunction (Rajagopalan et al., 2008). In a recent study, cardiomyocyte-specific ErbB1 downregulation in mice led to impaired contractility (Guo et al., 2021). Similarly, conditional deletion of its ligand, HB-EGF, also resulted in cardiac contractile defects (Iwamoto et al., 2003) implying EGFR/ErbB1 receptors play a critical role in maintaining contractile homeostasis in the adult heart.

Endogenous or exogenously administered EGF-like ligands (e.g., EGF, HB-EGF, betacellulin and amphiregulin) and subsequent activation of EGFR/ErbB1, is thought to play a beneficial cytoprotective role in the heart against stress induced injury (Lorita et al., 2002; Pareja et al., 2003). For example, acute, high intensity stress can cause cardiac damage via elevated catecholamine release and cardiomyocyte death by apoptosis and/or necrosis (Liaudet et al., 2014). Administration of EGF can prevent the damage caused by intense and sustained β-adrenergic stimulation and catecholamine release in the heart by interfering with β -adrenergic signaling (Lorita et al., 2002; Pareja et al., 2003). Further, it was shown that activated EGFR/ErbB1 plays a critical role in the cardiac protection against the acute, high intensity stress induced in fighting mice (Pareja et al., 2003) and may also offer protection against cardiac stressors during hibernation (Childers et al., 2019). During hibernation, animals experience a reduction in blood circulation that makes the heart prone to multiple stressors. Increased EGFR phosphorylation (Y1086) was found to play a key role in activation of MAPK signaling, inhibition of downstream p-ELK1, and reduction in both p-BAD mediated pro-apoptotic signaling and caspase-9 apoptotic signaling in the heart of hibernating ground squirrels (Childers et al., 2019). Activation of cardioprotective EGFR signaling likely explains how these mammals cope with cardiac stresses during hibernation, which otherwise can lead to serious cardiac injury (Childers et al., 2019).

To examine the physiological role of ErbB2 signaling in the adult heart, rats with a ventricular-specific deletion of ErbB2 were generated (Crone et al., 2002). After physiological analysis, they discovered that these mutant mice hearts showed wall thinning, chamber dilation and decreased contractility indicative of dilated cardiomyopathy (Crone et al., 2002). Thus, increasing expression or activation of ErbB2 might prevent dilated cardiomyopathy and heart failure accordingly. Further, in a model of type 1 diabetes, the effect of acute activation or chronic inhibition of EGFR and ErbB2 signaling on heart function was also studied (Akhtar et al., 2012a). Recovery of cardiac function following I/R injury in diabetic hearts was significantly impaired, most likely as a result of reduced dimerization and signaling by cardiac ErbB2 and EGFR receptors. Chronic administration of selective pharmacological inhibitors of either ErbB2 or EGFR/ErbB1 in diabetic animals exacerbated cardiac recovery. In contrast, acute stimulation of EGFR/ErbB2 signaling with EGF improved cardiac recovery following I/R injury (Akhtar et al., 2012a). These findings confirmed the beneficial role or EGFR and ErbB2 receptors and downstream signaling via ERK1/2, p38 MAP kinase and AKT in mediating recovery of cardiac function that is normally impaired in diabetes (Akhtar et al., 2012b).

In another study using in H9c2 rat cardiomyoblasts, pretreatment with EGF attenuated H_2_O_2_-induced oxidative stress and inhibited ROS-induced cell death and H_2_O_2_-induced apoptosis through activating Nrf2 (Ma and Jin, 2019). In animal models of myocardial I/R, *in vivo* administration of EGF diminished infarct size and myocardial apoptosis (Ma and Jin, 2019). Taken together, these data demonstrate that EGF attenuates oxidative stress and cardiac I/R injury by reducing myocardial infarct size and improving cardiac function possibly via activation of Nrf2 (Akhtar et al., 2012b; Ma and Jin, 2019).

During cardiac I/R injury, the myocardium becomes depleted of zinc and the capacity to mobilise labile zinc is reduced indicative of zinc dyshomeostasis. Administration of zinc pyrithione can normalize zinc levels during I/R and also prevents apoptosis by increasing ErbB1/ErbB2 levels (Bodiga et al., 2015) further confirming the cardioprotective role of these ErbB receptors. Zinc pyrithione attenuated caspase activation, decreased the proteolytic degradation of ErbB2, enhanced activation and complexation (heterodimerization) of ErbB1/ErbB2 that resulted in increased myocyte survival after hypoxia/reoxygenation injury (Bodiga et al., 2015).

EGFR/ErbB receptors also mediate the beneficial effects of cardiac preconditioning (Benter et al., 2005c; Akhtar et al., 2012b). It is well known that ischemic preconditioning, which typically involves exposure of the heart to brief episodes of ischemia-reperfusion (I/R), protects the myocardium from the greater damaging effects of subsequent more prolonged I/R. In a rat model of type 1 diabetes, chronic *in vivo* administration of a specific pharmacological inhibitor of EGFR abrogated the cardiac benefits of preconditioning implying a critical role of EGFR/ErbB1 in cardiac preconditioning (Akhtar et al., 2012b). In models of cardiac preconditioning induced by pharmacological agents, the cardioprotective effects of GPCR agonists such as bradykinin, A1 adenosine receptors, apelin, acetylcholine and a δ-opioid peptide were found to be also mediated via transactivation of EGFR (Krieg et al., 2004; Cohen et al., 2007; Methner et al., 2009; Williams-Pritchard et al., 2011; Folino et al., 2018). For example, apelin-induced-reduction in infarct size and myocardial contracture via its GPCR (termed APJ) were prevented by the inhibition of EGFR, Src, MMP or RISK (reperfusion injury salvage kinase) pathways (Folino et al., 2018). Recently, the cardioprotective effects of myocyte-specific hypoxia-inducible factor 2A were additionally reported to be mediated via EGFR (Lee et al., 2020) further implicating EGFR/ErbB1 receptors in a protective role during myocardial I/R injury. Moreover, the fact that a pan-ErbB inhibitor, genistein, also blocked the cardioprotective effects of I/R preconditioning (Akhtar et al., 2012b), implied that other ErbBs receptors, beyond EGFR, might also be involved in mediating the beneficial effects of cardiac preconditioning.

Previously it was thought that only EGFR, ErbB2 and ErbB4 were expressed in the adult heart but subsequent studies have demonstrated that post-natal cardiomyocytes of mice express a functional ErbB3 protein as well (Camprecios et al., 2011). More recently ErbB3 gene expression has also been reported in normal human ventricular cardiac fibroblasts (Kirabo et al., 2017). Although methylation of ErbB3 gene has been reported in human dilated cardiomyopathy (Haas et al., 2013); the function of ErbB3 in the adult heart is only now being understood. For example the E3 ligase (or neuregulin receptor degradation protein-1; Nrdp1), that selectively targets ErbB3 (Diamonti et al., 2002), is upregulated following I/R injury. Mice over-expressing Nrdp1 in the heart exhibit higher infarct size as well as increased apoptosis and inflammatory cell influx following I/R (Zhang et al., 2011) implying that Nrdp1 is a pro-apoptotic signal in the heart during I/R injury and exerts its actions via degradation of ErbB3. More recently it was reported that ErbB3 is a cardioprotective/pro-survival factor against redox stress (Morano et al., 2017). ErbB3 gene expression was transiently increased in the adult heart after I/R injury (Morano et al., 2017). However, I/R reduced ErbB3 protein levels, whereas postconditioning (that was induced by brief cycles of I/R immediately after ischemia) prevented I/R-induced reduction in ErbB3 receptor protein. Furthermore, the transient over-expression of ErbB3 gene alone was able to enhance cell survival and attenuate mitochondrial dysfunction and apoptosis in H9c2 cells exposed to redox-stress (Morano et al., 2017). This study implied that ErbB3 acts beneficially as a cytoprotective and/or pro-survival factor in the heart (Morano et al., 2017).

A cardiomyocyte-specific conditional ErbB4 KO mouse model was also used to prove that ErbB4 is required for remodeling of cardiomyocytes and essential in maintaining myocardial contractile function in the post-natal heart (Garcia-Rivello et al., 2005). Despite the normal heart morphology evident at birth, conditional ErbB4 KO mice developed severe dilated cardiomyopathy and conduction abnormalities that lead to premature death (Garcia-Rivello et al., 2005).

The HB-EGF ligand binds to and triggers signaling via several ErbB receptors including EGFR/ErbB1 and ErbB4 receptors. In experiments with HB-EGF-null mice, over half of the mice lacking this ligand died within a week. The rest showed severe heart failure, enlarged cardiac valves and ventricular chambers with a resultant impairment of cardiac function. In contrast, administration of HB-EGF in WT mice increased phosphorylation of cardiac ErbB2, ErbB4, and to a lesser degree, of EGFR (Iwamoto et al., 2003) implying that HB-EGF-induced activation of multiple ErbB receptors is crucial for normal heart function.

The cardiac effects of NRG-1, another EGF-like ligand, have been extensively studied. The stimulation of ErbB signaling by NRG-1 appears crucial for cardiomyocyte survival and furthermore, is an important compensatory mechanism in the failing adult heart (De Keulenaer et al., 2019). Neuregulin ligand-mediated ErbB receptor signaling plays a key role in cellular functions such as proliferation, differentiation, migration and protection against cell death through controlling bcl-x splicing and bcl-2 family protein expression, as well as activation of the mammalian target of rapamycin (mTOR) that induces protein synthesis and hypertrophy (for reviews see Sanchez-Soria and Camenisch, 2010; De Keulenaer et al., 2019). Thus, through multiple effectors, NRG-1 induces pro-survival gene expression and enhances proliferation and survival of human cardiac fibroblasts (Fang et al., 2017; Kirabo et al., 2017; De Keulner et al., 2019). For example, NRG-1 counters the upregulation of endoplasmic reticulum (ER) stress markers like glucose-regulated protein 78 and cleaved caspase-12 in ventricular myocytes through ErbB4 receptors. Inhibition of EGFR/ErbB1 signaling or ErbB4 knockdown reversed the beneficial effects of NRG-1 in inhibiting ER stress in cultured neonatal cardiomyocytes (Fang et al., 2017). Moreover, in an *in vivo* rat model of cardiac I/R injury, intravenous NRG-1 administration significantly decreased ER stress and myocardial infarct size (Fang et al., 2017). Thus, NRG-1 has a protective role in I/R injury by inhibiting myocardial ER stress that is likely mediated by several ErbB receptors including ErbB4. Indeed, these pro-survival effects of NRG-1 have led to its consideration as a potential therapeutic agent for treatment of heart failure in pre-clinical and human clinical studies that have collectively shown that i. v., administration of this ligand significantly improved cardiac contractility and remodelling as well as attenuated mitochondrial dysfunction and apoptosis (Liu et al., 2006; Gao et al., 2010; Jabbour et al., 2011; Guo et al., 2012; Xiao et al., 2012; Hill et al., 2013; De Keulenaer et al., 2019). Additionally, and of relevance to the diabetic heart, is the potential glucose-lowering ability of NRG-1. Acute administration of this ligand decreased the glycaemic response to an oral glucose tolerance test in rats (Caillaud et al., 2016) implying that it might have added therapeutic value as a glucose-lowering agent in diabetic patients with heart failure.

Interestingly, monoclonal antibody and small molecule-based inhibitors of EGFR/ErbB receptors are now extensively used in the treatment of cancer (Roskoski, 2019; Kumagai et al., 2021). However, their use may lead to unwanted cardiac toxicities ranging from reduced LV ejection fraction to heart failure (Kenigsberg et al., 2017; Cuomo et al., 2019). In an early study, female (but strangely, not male) mice chronically administered either of two EGFR small molecule tyrosine kinase inhibitors, lead to physiological and pathological cardiac changes such as altered LV wall thickness and increased apoptosis (Barrick et al., 2008). Using a human-induced pluripotent stem cell-derived cardiomyocyte *in vitro* model, treatment with trastuzumab, a monoclonal antibody inhibitor of ErbB2, also aggravated cardiomyocyte damage, whereas activation of ErbB signaling with NRG-1 alleviated cardiomyocyte injury (Eldridge et al., 2014). In the clinic, trastuzumab is known to exhibit cardiac toxicity particularly in patients with abnormal LV function but cardiotoxicity with other ErbB inhibitors such as gefitinib (a small molecule EGFR-specific inhibitor) or even lapatinib (a dual EGFR-ErbB2 inhibitor) appears to be rare (Kenigsberg et al., 2017). Thus, studies on the cardiotoxicity of some ErbB receptor inhibitors are supportive of a cardioprotective role of ErbBs in the adult heart. However, since not all ErbB inhibitors exhibit cardiotoxicity, the possibility that the observed adverse effects on the heart might be due to other “off-target”, but drug-specific, effects remains to be elucidated.

### The Detrimental Role of Epidermal Growth Factor Receptor in the Heart

The studies described in the preceding section (see also Table 1) clearly suggest a beneficial and necessary role of EGFR/ErbB/HER receptors in the developing heart. They further imply that acute or transient activation of EGFR/ErbB/HER receptor TKs and subsequent downstream activation of survival (or salvage/recovery) pathways such as PI3K/AKT/mTOR and RISK is required for mediating beneficial cardioprotective effects in I/R injury. In contrast, there is now a growing body of evidence that supports the notion that chronic or persistent overexpression/activation of EGFR/ErbB family of receptors can play a detrimental role (see Table 2) in the pathological heart including in cardiac hypertrophy, fibrosis and cardiac remodeling associated with diabetes (e.g., Kobayashi and Eguchi, 2012). As EGFR/ErbB/HER receptors are known drivers of cell growth and proliferation via mitogenic signaling through Ras/Raf/MAP kinases, AKT and ERK1/2, they play key role in mediating cardiac hypertrophy (Peng et al., 2016; Bi et al., 2020). For example, Ang II-mediated cardiac hypertrophy is reported to occur via Src-dependent EGFR transactivation through AKT and ERK pathways that can be reversed with pharmacological inhibitors of EGFR (Liang et al., 2015; Peng et al., 2016). In addition to Ang II, other members of the RAAS can also transactivate EGFR/ErbB receptors to impact cardiac function. In this regard, EGFR transactivation is known to be involved in aldosterone-induced NHE-1 (Na^+^/H^+^ Exchanger 1) stimulation that mediates oxidative stress and subsequent cardiovascular damage (De Giusti et al., 2011).

**TABLE 2:** A summary of selected *in vivo* studies highlighting the **detrimental** role of EGFR/ErbB signaling in the heart.

Role of EGFR	Study model	Intervention	Results	Refs.
**Amphiregulin via EGFR activation increases cardiac fibrosis after myocardial infarction**	C57BL6 mice	Amphiregulin and gefitinib	• Amphiregulin, via EGFR activation, promoted cardiac fibroblast migration, proliferation, and collagen synthesis (gefitinib abrogated all these effects)	Liu et al. (2020)
			• Amphiregulin deletion improved cardiac function and increased survival rate	
**EGFR contribute to myocardial infarction in T2D**	T2D mice (db−/db−)	Group 1: Untreated	• EGFR and ER stress inhibitors reduced the cell apoptosis, inflammation, and myocardial infarct size in T2D mice after myocardial I/R injury induction	Mali et al. (2018)
		Group 2: AG1478		
		Group 3: Tudca		
**EGFR plays a role cardiac fibrosis**	C57Bl/6J mice	Generation of osteoglycin-null mice (OGN-/-)	• OGN, via EGFR inhibition, negatively regulated cardiac fibrosis by attenuating myofibroblast proliferation and migration	Zuo et al. (2018)
		Ang II infusion	• Chronic Ang II infusion in OGN deficient mice increased cardiac fibrosis and impaired cardiac function	
**EGFR increases cardiac remodeling in diabetic cardiomyopathy **	C57/BL6 mice	Gefitinib and ramipril	• Gefitinib, via EGFR inhibition, prevented lipid peroxidation, antioxidant enzymes damage, myocardial hypertrophy, myocardial damage and improved Ca^2+^ homeostasis in STZ-induced cardiomyopathy	Shah et al. (2018)
**EGFR induces cardiac hypertrophy**	C57BL/6 mice	AG1478, 542 and 54	• AG1478, 542 and 543, via EGFR inhibition, attenuated ang II- and EGF-induced cardiac hypertrophy	Peng et al. (2016)
**EGFR contributes to cardiac inflammation and injury associated with high fiber diet**	Apo-E knockout mice	AG1478 and 542	• EGFR inhibitors attenuated palmitic acid and hyperlipidemia-induced cardiac injury and inflammation in mice fed with high fat	(Li et al., 2016)
**EGFR role in cardiac damage and remodeling through oxidative stress**	STZ-induced T1D mice	AG1478 and 451	• AG1478 and 451, via EGFR inhibition, decreased diabetes-induced oxidative stress, cardiac remodeling, hypertrophy, fibrosis and apoptosis	Liang et al. (2015)
**EGFR and its downstream ER stress lead to cardiac injury and microvascular dysfunction in T1D**	C57BL/6J	STZ only or in combination with AG1478, tudca or insulin	• AG1478, tudca, and insulin reduced cardiac fibrosis, collagen type I, and plasminogen activator inhibitor 1 and restored the impaired epithelium dependent and independent relaxation responses in T1D mice	Galan et al. (2012)
**EGFR contributes to ROS production and cardiac damage**	Wistar rats	Aldosterone and EGF	• Aldosterone-induced NHE-1 stimulation, via EGFR transactivation resulted in ROS production and cardiac injury	De Giusti et al. (2011)

T2D, type 2 diabetes; ER, endoplasmic reticulum; I/R, ischemia/reperfusion; Ang II, angiotensin II; STZ, streptozocin; ApoE, apolipoprotein E; ROS, reactive oxygen species; NHE, sodium hydrogen exporter.

Hyperglycemia associated with diabetes is known to lead to the generation of reactive oxygen species (ROS) such as via NADPH oxidase (NOX) activity (see also *The Link Between Epidermal Growth Factor Receptor & Nicotinamide Adenine Dinucleotide Phosphate Oxidase in Diabetes-Induced Vascular Dysfunction*) that can lead to cardiovascular complications (Brownlee, 2005; Zhang et al., 2020; Babel and Dandekar, 2021). There is emerging evidence that EGFR inhibitors can prevent ROS formation and development of oxidative stress that leads to cardiac dysfunction (Liang et al., 2015; Shah et al., 2018). EGFR inhibition with gefitinib decreased hyperglycemia-induced cardiac remodeling by preventing oxidative stress-induced changes in the diabetic heart (Shah et al., 2018). Administration of gefitinib (a selective EGFR inhibitor) in C57/BL6 mice, significantly prevented lipid peroxidation and damage of antioxidant enzymes like glutathione and thioredoxin reductase. It also prevented myocardial hypertrophy and attenuated the diabetes-induced alterations in collagen deposition and myocardial remodeling. Administration of gefitinib also prevented depletion of SERCA2a (sarcoplasmic endoplasmic reticulum Ca^2+^ ATPase2a) and NCX1 (sodium-calcium exchanger-1) in streptozotocin-induced cardiomyopathy-indicative of improved Ca^2+^ homeostasis during myocardial contractility (Shah et al., 2018). These findings suggest that inhibition of EGFR can reduce cardiac damage in diabetic cardiomyopathy through balancing oxidant-antioxidant systems and attenuating subsequent hypertrophy and remodeling in the diabetic heart. Thus, gefitinib, and other clinically approved EGFR inhibitors that do not exhibit cardiotoxicity, may be considered for the potential treatment of diabetic cardiomyopathy.

Emerging evidence supports the view that generation of ROS and oxidative stress is strongly associated with the occurrence of endoplasmic reticulum (ER) stress (Chong et al., 2017). The ER is responsible for the correct folding, processing and trafficking of secretory and membrane-bound proteins to the Golgi apparatus, but when the capacity of this sub-cellular organelle to fold proteins becomes saturated, such as in oxidative stress, ER stress ensues that can lead to multiple pathologies (Navid and Colbert, 2017) including diabetes-induced cardiovascular complications (Xu et al., 2012; Sankrityayan et al., 2019). In a mouse model of type 1 diabetes, upregulation of EGFR tyrosine kinase led to induction of ER stress that resulted in diabetes induced cardiovascular dysfunction (Galan et al., 2012). Pharmacological inhibition of EGFR attenuated receptor phosphorylation and reduced ER stress, cardiac fibrosis, and microvascular dysfunction (Galan et al., 2012) implying a key role of EGFR in mediating ER stress and subsequent cardiac pathology. The detrimental role of EGFR in the pathogenesis of diabetes-induced heart damage was further confirmed by Liang et al. (2015). They reported that Ang II-induced cardiac hypertrophy, fibrosis and remodeling was mediated via enhanced signaling through a Src-dependent EGFR/AKT pathway. The fact that significant attenuation of these pathological changes occurred after treatment with gene silencing shRNAs or EGFR inhibitors provided strong evidence that aberrent EGFR signaling plays a crucial role in the pathogenesis of cardiac dysfunction and remodeling in diabetes (Liang et al., 2015). Additionally, EGFR signaling and ER stress are also reported to play a role in inducing myocardial infarction in type 2 diabetes (Mali et al., 2018). To investigate this role, T2D mice were treated with AG1478 (a specific EGFR inhibitor) or TUDCA (an ER stress inhibitor) for 2 weeks. After that, acute myocardial I/R injury was induced. Treatment with EGFR and ER stress inhibitors in T2D mice significantly reduced the extent of the myocardial infarct size compared to untreated T2D mice. Inhibition of EGFR and ER stress was associated with reduced myocardium p38 and ERK1/2 MAP-kinases activity, increased pro-survival signaling via AKT, and reduced inflammatory cell infiltration and apoptosis (Mali et al., 2018). By suggesting that EGFR signaling was detrimental in acute myocardial I/R injury, this study (Mali et al., 2018) was at variance with multiple other reports showing that EGFR signaling is beneficial in the recovery of cardiac function following I/R injury (Ma and Jin 2019; Akhtar et al., 2012b; Pareja et al., 2003; Lorita et al., 2002). The precise reasons for this discrepancy are not known but the use of different animal models and experimental conditions may be possible explanations. For example, Mali et al. (2018) used a T2DM (db^−^/db^−^) mouse model with regional ischemia compared to a T1DM (streptozotocin-induced) rat model with global ischemia used by Akhtar et al. (2012b). How these potential variables precisely impact on EGFR/ErbB signaling requires further study. Nonetheless, collectively these studies suggest that targeting of EGFR and ER stress may represent novel therapeutic strategies in the potential treatment of diabetes-induced cardiac pathologies.

Obesity is a leading risk factor for the development of type 2 diabetes and its cardiovascular complications (Leitner et al., 2017; Drucker, 2021). In a study aiming to determine if EGFR mediates the pathogenesis of hyperlipidemia/obesity-related cardiac diseases, EGFR inhibition significantly ameliorated myocardial fibrosis, apoptosis, and inflammation in two rat models of obesity (Li et al., 2016). Thus, consistent with reports in models of diabetes, this study also demonstrated a harmful effect of EGFR activation in the pathogenesis of obesity-induced cardiac pathology (Li et al., 2016) and thus, potential use of EGFR inhibitors for the treatment for obesity-related cardiovascular complications may be warranted.

EGFR signaling is additionally thought to be important in the cardiac actions of osteoglycin (OGN), a small leucine-rich proteoglycan that also regulates bone and glucose homeostasis (Lee et al., 2018; Zuo et al., 2018). OGN negatively regulates cardiac fibrosis by attenuating cardiac myofibroblast (CMF) proliferation and migration through inhibition of EGFR signaling. Thus, OGN deficiency promoted EGFR and ERK1/2 phosphorylation in CMFs cultured from adult and neonate mice. In contrast, restoration of OGN in OGN-null CMFs resulted in inhibition of cell migration and proliferation through decreased EGFR signaling (Zuo et al., 2018). Stimulation of EGFR/ErbB receptors by amphiregulin (AR), another member of EGF family of ligands, induced cardiac fibrosis after myocardial infarction (Liu et al., 2018; Liu et al., 2020) highlighting the detrimental role of EGFR/ErbBs in cardiac pathology. EGFR transactivation was also involved in mediating the non-canonical, cardiomyocyte hypertrophy-promoting, effects of the death receptor 5 that normally mediates apoptosis (Tanner et al., 2019). Interestingly a very recent study showed that increased EGFR signalling in the rat brain, namely within the hypothalamic paraventricular nucleus (PVN), may also play a key role in mediating myocardial-infarction-induced heart failure through enhancing ERK1/2-induced sympathetic overactivity (Yu et al., 2021). Selective gene silencing of EGFR in the PVN led to a correction of multiple central and peripheral markers associated with heart failure (Yu et al., 2021) implying that targeting brain EGFR might represent a novel approach in the treatment of MI-induced HF. Collectively, the above studies suggest a critical role for both peripheral and central EGFR/ErbB signaling in the development of cardiac pathologies, and further imply that these RTKs may serve as a “hub” or “relay” for other signaling molecules/inputs that induce cardiac complications in diabetes.

**FIGURE 1:**
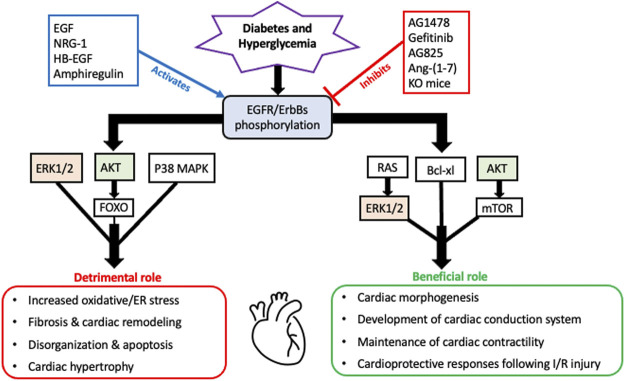
A schematic highlighting the overall beneficial and detrimental actions of EGFR/ErbB receptors and their downstream signaling effectors in the diabetic heart. It appears that all four EGFR/ErbB receptors are necessary for the development of the heart during embryogenesis and beneficial for the proper functioning of the adult heart including a key role in maintenance of cardiac contractility. EGFR/ErbB2 signaling is also essential for normal cardiomyocyte function. Acute stimulation of EGFR/ErbB receptor signaling is also essential for activation of salvage pathways and cardiac recovery following I/R injury. In addition to these beneficial actions, EGFR/ErbB receptor signaling can also have detrimental outcomes. Persistent or chronic upregulation of EGFR/ErbB receptor signaling has been involved in the development of cardiac dysfunction and pathologies including fibrosis, remodelling and hypertrophy. Several key downstream effectors are involved in mediating both actions with some notable overlaps such as AKT and ERK1/2 (highlighted)- implying that they too can have dual actions depending on the pathophysiological context akin to the role of their upstream EGFR/ErbB receptors (see main text for further details and references).

As a summary of their cardiac actions, Figure 1 highlights the overall beneficial and detrimental actions of EGFR/ErbB2/HER receptors and downstream effector signaling in the heart (see also perspectives and concluding remarks section below).

## Diabetes-Induced Vascular Dysfunction

Both T1DM and T2DM can lead to potentially life-threatening microvascular and macrovascular complications such as retinopathy, neuropathy, nephropathy, as well as coronary heart disease and stroke (Kosiborod et al., 2018; Colom et al., 2021). Similar to that described in the heart (see above), diabetes-induced vascular complications generally result from chronic hyperglycemia (Van Wijngaarden et al., 2017) though repeated transient excursions into hyperglycemia and glycemic variability also increase the risk of developing vascular complications (Holman et al., 2008; Gray and Jandeleit-Dahm, 2014; Kenny and Abel, 2019; Babel and Dandekar, 2021). Correction of hyperglycemia in diabetic patients also does not completely restore the risk of developing vascular complications to baseline (Ceriello, 2005; Brownlee and Hirsch, 2006; Chalmers and Cooper, 2008; Kenny and Abel, 2019) implying that hyperglycemia-induced vascular changes, most likely at a molecular level, are long-lasting–a phenomenon termed “glycemic” or “metabolic memory”. Although there appears to be some differences in the pathology of micro-and macro-vascular dysfunction (e.g., the latter is most common in T2DM which comprises around 85–90% of all diabetic patients), both involve damage to the endothelium, the innermost lining of blood vessels, that is involved in many important vascular functions including maintenance of vascular tone (Shi and Vanhoutte, 2017); indeed, endothelial dysfunction represents a significant underlying cause of organ dysfunction and failure in several pathologies including diabetes and SARS-CoV2 (COVID19) infections (Shi and Vanhoutte, 2017; Jin et al., 2020; Nagele et al., 2020). In the vasculature, key dysfunctional changes of the tunica media such as increased vascular smooth muscle responsiveness to endothelium-dependent vasoconstrictors, are early features of diabetic vasculopathy (Cooper et al., 2001; Lee et al., 2012). Since diabetes-induced cardiac dysfunction also involves endothelial dysfunction, it is not surprising that endothelial dysfunction associated with micro- or macro-vascular complications most likely arises from a similar, though not necessarily identical, concoction of underlying biological mechanisms. Of note in the diabetic vasculature are increased intracellular Ca^2+^, diminished NO synthesis, increased polyol pathway flux, altered cellular redox state (ROS/oxidative and ER stress), prostanoid synthesis, accelerated nonenzymatic formation of advanced glycation end products (AGEs), increased formation of diacylglycerol and activation of specific protein kinase C (PKC) isoforms, inflammation and enhanced apoptosis (for review see Babel and Dandekar, 2021; Shi and Vanhoutte, 2017; Forbes and Cooper, 2013, Giacco and Brownlee, 2010; Brownlee, 2005). Since endothelial and vascular smooth muscle (VSM) cells (VSMCs) are closely linked functionally, many of the molecular pathways effecting endothelial dysfunction are also altered in VSMCs implying that a combination of endothelial and smooth muscle dysfunction is ultimately responsible for the abnormalities of vascular function in diabetes. Indeed, in patients with T2DM, VSM dysfunction was noted in both the micro- and macro-vasculature but was more pronounced in the microvasculature-i.e. blood vessels with diameter of  < 150 µm (Montero et al., 2013). In terms of mechanisms, in addition to ROS generation and oxidative stress, diabetes-induced alterations in signaling by growth factors, RTKs, peptides of RAAS as well as other GPCRs that exert significant influence on intracellular calcium levels, ROS, ER stress, inflammation and apoptosis appear to be involved in mediating VSMC dysfunction (Babel and Dandekar, 2021). For example, signalling via vascular endothelial growth factor (VEGF) appears important in mediating retinopathy in diabetes (reviewed in Wong et al., 2016), and hyperglycemia-induced up-regulation of GPCR signalling by the RAAS peptide, Ang II, has been reported in VSMCs (Montezano et al., 2014; Touyz et al., 2018). Since it is now known that GPCRs can initiate transactivation of the EGFR (e.g., Akhtar et al., 2015; Forrester et al., 2016), which is expressed in both endothelial cells and VSMCs (e.g., Akhtar et al., 2012a; Schreier et al., 2021), there is strong indications that EGFR signalling may also be a key player in mediating vascular pathology in diabetes (Akhtar and Benter, 2013; Matrougui, 2010; Schreier et al., 2014; Schreier et al., 2018).

### Role of Epidermal Growth Factor Receptors in the Diabetic Vasculature

EGFR/ErbB receptor signaling that typically regulates cell growth, differentiation and proliferation is essential to the normal physiologically development and functioning of the vasculature (Forrester et al., 2016). However, its chronic dysregulation appears to have a detrimental role in mediating diabetes-induced vascular dysfunction and remodelling (Benter et al., 2005; Akhtar and Benter, 2013; Matrougui, 2010; Akhtar et al., 2019; Schreier, et al., 2014; Schreier et al., 2018) see also Table 3.

**TABLE 3:** A summary of selected studies implicating EGFR signaling in diabetes-induced vascular dysfunction.

Role of EGFR	Study model	Intervention	Results	Refs.
**Endothelial EGFR compared to vascular smooth muscle (VSM) EGFR plays a minor role in obesity/DIVD**	High-fat diet T2DM mice	Endothelial cell (EC) specific EGFR knockout	• A comparison of vascular function parameters in EC verus VSM-specific EGFR KO mice implied that EC-EGFR plays a minor role in mediating obesity/T2DM-induced vascular dysfunction	Schreier et al. (2021)
**Vascular smooth muscle (VSM) specific deletion of EGFR prevents obesity and DIVD**	High-fat diet T2DM mice	VSM-specific EGFR-knockout (KO)	• VSM-specific EGFR via ErbB2- ROCK–MRTF–SRF signalling mediates vascular dysfunctiom	Stern et al. (2020)
**DIVD and vascular remodeling can be corrected by inhibition of EGFR by novel polymeric nanoparticles**	STZ-induced T1DM wistar rats	PAMAM dendrimers	• AG1478 or PAMAMs (novel inhibitors of ErbBs) corrected abnormal vascular reactivity and vascular remodeling in diabetic MVB	Akhtar et al. (2019)
		AG1478		
**Multiple downstream pathways are involved in EGFR- ErB2 mediated DIVD**	STZ-induced T1DM wistar rats	Lapatinib	• lapatinib inhibits EGFR & ErbB2 activity in diabetic MVB and in VSMCs and reverses diabetes and hyperglycemia-induced changes in ERKs, ROCK, AKT, FOXO, eNOS, & NF-kB and prevents HG-induced apoptosis in VSMCs	Benter et al. (2015)
**Src-dependent EGFR/ErbB2 transactivation via ang II leads to DIVD**	STZ-induced T1DM wistar rats	Ang-(1–7)	• Ang-(1–7) inhibits diabetes-induced transactivation of ErbB receptors in isolated MVB and opposes downstream signaling changes in ERK1/2, p38 MAPK, ROCK, eNOS, and lkB-α	Akhtar et al. (2015)
**Increased EGFR activity via NADPH-oxidase leads to DIVD**	C57/BL6 and db^−^/db^−^ T2D mice	AG1478, or exogenous EGF, or gp-91 ds-tat	• T2DM or EGF alters vascular reactivity in MRA that could be corrected by AG1478. EGF increased ROCK expression in MRA that could be corrected by NOX inhibition	Kassan et al. (2015a)
**EGFR-ErbB2 heterodimers via ERK1/2- ROCKs lead to DVID**	STZ-T1DM wistar rats	AG825	• Chronic *in vivo* or acute *ex vivo* inhibition of ErbB2 or EGFR corrected DIVD in MVB and attenuated elevated ERK1/2 and ROCK signaling. First evidence for EGFR-ErbB2 heterodimerization presented in co-association/immunoprecipitation assays	Akhtar et al. (2013)
		Anti-ErbB2 siRNA		
		AG1478		
**Elevated EGFR mRNA an early change in development of DIVD**	STZ-T1DM wistar rats	AG1478	• Diabetes altered expression of over 1,300 genes in MVB. AG1478 treatment prevented 95% of these changes implying that EGFR is a key early change in the development of DIVD	Benter et al. (2009)
**Elevated EGFR via reduced eNOS mediates DIVD**	T2DM db/db mice	AG1478	• Altered vascular reactivity and reduced eNOS expression in MRA and CA of diabetic mice could be corrected by AG1478 treatment	Belmadani et al. (2008)
**EGFR and ErbB2 mediate DIVD in carotid artery**	STZ-T1DM wistar rats	AG1478	• Altered vascular reactivity in diabetic carotid artery could be corrected by AG1478 and AG825 implicating EGFR and ErbB receptors in mediating DIVD	Yousif et al. (2005)
		AG825		
**Increased EGFR signaling leads to DIVD in renal artery**	STZ-T1DM wistar rats	AG1478	• Elevated EGFR and DIVD in renal artery could be corrected by AG1478 and genistein but not by diadzein	Benter et al. (2005b)
		Genistein diadzein		
**Elevated EGFR phosphorylation leads to DIVD in MVB**	STZ-T1DM wistar rats	AG1478	• Diabetes-induced abnormal vascular reactivity in MVB was linked to increased EGFR phosphorylation that could be corrected by AG178 treatment	Benter et al. (2005a)
		Genistein		

STZ, streptozocin; MVB, mesenteric vascular bed; VSMC, vascular smooth muscle cell; HG, hyperglycemia; MRA, mesenteric resistance arteries, CA, coronary artery; DIVD, Diabetes induced vascular dysfunction; AG1478, A selective EGFR Inhibitor; AG825, A selective ErbB2 inhibitor; lapatanib, A dual EGFR and ErbB2 inhibitor; Genistein, A pan ErbB/tyrosine kinase inhibitor; Diadzein, an inactive analogue of genistein; Ang-(1–7), Angiotensin-(1–7); gp91 das-tat, specific inhibitor of NADPH oxidase (NOX); PAMAM, Polyamidoamine (as dendrimeric nanoparticles); ROCK, Rho kinase; MRTF, actin–myocardin-related transcription factor; SRF, serum response factor.

Probably the early clues to the involvement of EGFR/ErbB receptors in diabetes-induced complications were the observation that excretion of EGF ligand was abnormal in many patients with diabetes (Lev-Ran et al., 1990) and that EGF gene expression was elevated in the mesenteric artery of rats bearing type 1 diabetes (Gilbert et al., 2000). Further, since vascular proliferation and remodeling (e.g., via hypertrophy, hyperplasia and fibrosis) leads to altered vascular contractility in diabetes, of note were the studies showing a pro-contractile role for EGF/EGFR signaling (Berk et al., 1985; Florian and Watts, 1999). For example, EGF via stimulation of EGFR elicited potent vasoconstriction in aortic strips from a rat model of hypertension (Florian and Watts, 1999); and EGFR activation via MMP-2 reportedly increased ROS formation and facilitated contraction in non-diabetic aortas (Prado et al., 2018). Additionally, there is also evidence in the non-diabetic vasculature that acute EGFR signaling may mediate the contractions of GPCR ligands such as Ang II and endothelin (ET-1) (Kawanabe et al., 2004; Lucchesi et al., 2004) that also play an important role in mediating diabetes-induced vascular dysfunction. A recent study also suggests that dysregulation of vascular, as opposed to endothelial, EGFR might be the dominant factor in the development of vascular pathologies associated with obesity (Schreier et al., 2021) an important risk factor for developing T2DM.

The first *in vivo* reports demonstrating a direct link between EGFR signaling and diabetes-induced vascular dysfunction appeared in 2005 which showed that enhanced EGFR/ErbB1 signaling was a key mediator of diabetes-induced vascular dysfunction as pharmacological inhibition of this RTK was corrective of this pathology in the mesenteric vascular bed as well as in renal and carotid arteries (Benter et al., 2005a; Benter et al., 2005b; Yousif et al., 2005). This initial research on vascular EGFR was conducted in a preclinical model of type 1 diabetes (streptozotocin-induced diabetes in rats) and its findings were subsequently replicated in the vasculature of animal models of type 2 diabetes (Belmadani et al., 2008; Palen and Matrougui, 2008; Choi et al., 2012; Galan et al., 2012; Kassan et al., 2015a; Stern et al., 2020), implying that dysregulated EGFR/ErbB1/HER1 signaling was a common mediator of vascular complications in both Type 1 and Type 2 diabetes. Further, through gene expression profiling studies of diabetic mesenteric vasculature, it was demonstrated that EGFR1/ErbB1/HER1 inhibition could normalize approximately 90% of the transcriptomic changes that occurred during the development of diabetes-induced vascular abnormalities (Benter et al., 2009). In addition, increased gene expression of EGFR1/ErbB1/HER1 appeared to be a critical early mRNA change resulting in diabetes-induced vascular dysfunction (Benter et al., 2009). Studies from the same group on the underlying mechanisms, identified that EGFR1/HER1 did not act alone, rather heterodimerization with ErbB2 (its preferred dimerization partner) and subsequently signaling via multiple pathways including PI3K/AKT were key mediators of diabetes-induced vascular complications (Akhtar et al., 2013; Akhtar et al., 2015). More recently, these authors showed that all ErbB receptors are activated by hyperglycemia (Akhtar et al., 2015; Akhtar et al., 2020b) and that pan-ErbB inhibitors can reverse these changes and correct vascular dysfunction in a rat model of type 1 diabetes (Akhtar et al., 2020b; Akhtar et al., 2019) implying a possible role for all EGFR/ErbB receptors in mediating diabetes-induced vascular dysfunction.

Src family of non-receptor tyrosine kinases appear to be key upstream effectors of EGFR/ErbB RTKs as hyperglycemia/diabetes is known to induce EGFR/ErbB signaling via a Src-dependent mechanism (Akhtar et al., 2012a; Akhtar et al., 2015; see also T*he Interplay of Epidermal Growth Factor Receptor and Angiotensin 1-7, a Member of the Renin-Angiotensin-Aldosterone System, in Diabetes-Induced Vascular Dysfunction* below). As to the downstream effectors of EGFR/ErbB signaling in the diabetic vasculature, several studies have now implied that multiple downstream pathways are likely involved including dysregulation of NO, Rho kinases (ROCKs), ERK1/2 and PI3K/AKT pathways (Akhtar and Benter, 2013; Benter et al., 2015; Matrougui, 2010; Schreier et al., 2014; Schreier et al., 2018). For example, diabetes enhances phosphorylation of EGFR1 and ErbB2, formation of EGFR/ErbB2 heterodimers and subsequent elevation in ERK1/2 and ROCK signaling leading to dysfunction in the mesenteric vasculature of T1DM rats (Akhtar et al., 2013; Benter et al., 2015). Administration of AG1478, a selective EGFR inhibitor, or AG825, a specific ErbB2 inhibitor, or Fasudil, a ROCK inhibitor, or PD98059, an ERK1/2 signaling inhibitor, all attenuated the observed changes associated with diabetes-induced vascular dysfunction (Akhtar et al., 2013) indicating the importance of these effectors in this pathology. Similarly, in a mouse model of T2DM, EGFR inhibition rescued abnormal ROCK activity and restored vascular function (Kassan et al., 2015b). Collectively, these results implied that EGFR/ErbB2-ERK1/2-ROCK pathway is an important mediator of diabetes-induced vascular dysfunction. The role of ROCK as a downstream effector of EGFR/ErbB2 heterodimers was also confirmed recently in a mouse model of high-fat diet induced obesity/type 2 diabetes bearing vascular smooth muscle-specific deletion of EGFR (Stern et al., 2020). This study showed that EGFR expressed in vascular smooth muscle cells mediates vascular remodeling via a ROCK-dependent activation of serum response factor - a transcription factor that regulates cell growth and proliferation (Stern et al., 2020). Interestingly, administration of the ROCK inhibitor, Fasudil, in the early stages of experimental diabetes was found to markedly suppress vascular hyperreactivity, increase tissue perfusion, and prevent organ damage whereas late-stage Fasudil administration had little or no effect (Li et al., 2015). This observation is suggestive of a threshold of vascular damage beyond which recovery of function is limited with ROCK inhibitors (Li et al., 2015) as well as possibly with other types of pharmacological interventions. Also of interest here is that EGF-ligand induced vasoconstriction is dependent on ROCK signaling. In rat aortic rings, EGF induced Ca^2+^ sensitization of VSMCs via a Rho-kinase-dependent inactivation of myosin light chain phosphatase (MLCP) mediated by the EGFR-MEK-ERK1/2 signaling pathway (Sasahara et al., 2015). Taken together, these studies suggest an important role of hyperglycemia-induced Src-dependent transactivation of EGFR/ErbB receptors and a downstream ROCK-dependent pro-contractile action in mediating diabetes-induced vascular dysfunction.

### Endothelial Nitric Oxide Synthase as an Effector of Epidermal Growth Factor Receptor or ErbB2 in Diabetes-Induced Vascular Dysfunction

Endothelial and thereby vascular dysfunction is characterised by a decreased production of nitric oxide (NO). In the vasculature, NO is mainly produced from l-arginine by endothelial nitric oxide synthase (eNOS) in a Ca^2+^/calmodulin-dependent protein kinase II (CaMKII) dependent reaction (Forstermann and Sessa, 2012; Zhao et al., 2015; Murthy et al., 2017). NO-mediated signaling is fundamental in maintaining vascular function as it can potentially prevent vascular inflammation, thrombosis, and cell proliferation (Bian et al., 2008; Zhao et al., 2015). eNOS activity is controlled through multi-site phosphorylation. For instance, enhanced phosphorylation of eNOS at Thr495 and decreased phosphorylation at Ser1177 site in the vascular endothelium leads to inadequate NO synthesis and impaired endothelium-dependent relaxation (Long et al., 2007). Diabetes reduces eNOS Ser1177 phosphorylation, thereby decreasing NO levels in endothelial cells and VSMCs (Tousoulis et al., 2012; Benter et al., 2015). Additionally, several studies have now determined that eNOS is a downstream effector of EGFR or ErbB2 signaling in T1DM and T2DM (Belmadani et al., 2008; Galan et al., 2012; Benter et al., 2015), and that exaggerated ErbB2-EGFR heterodimerization in the diabetic vasculature leads to diminished NO activity (Benter et al., 2015). Administration of lapatinib, which is a dual inhibitor of EGFR and ErbB2 RTKs, ameliorates vascular dysfunction by normalizing the decreased NO levels in the diabetic vasculature (Benter et al., 2015). Regarding restoration of NO activity in the diabetic vasculature by selective EGFR inhibitors, studies have shown that chronic *in vivo* AG1478 treatment rescued eNOS phosphorylation and expression (Belmadani et al., 2008; Galan et al., 2012) whereas acute *in vitro* AG1478 treatment appeared not to (Kassan et al., 2015a). It is worth mentioning that ROS generation by NADPH oxidase accounts partly for the reduced NO bioavailability in the vascular bed (Kassan et al., 2015a), consequently inducing dysfunction of the vascular endothelium (Sena et al., 2008; Akar et al., 2011; Meyrelles et al., 2011). Interestingly, a CaMKII inhibitor, KN-93, was also able to prevent the development of abnormal vascular reactivity in a rat model of diabetes and hypertension (Yousif et al., 2008) implying that NO production might also involve CaMKII-independent pathways in such pathologies.

### PI3K as an Effector of Epidermal Growth Factor Receptor in Diabetes-Induced Vascular Dysfunction

PI3Ks are lipid kinases that belong to a group of enzymes involved in the regulation of multiple signaling cascades that control multiple cellular processes including cell growth, proliferation, motility, and survival. It is well known that PI3K/AKT signaling is downstream of EGFR/ErbB signalling (Roskoski 2019) and also can activate eNOS and subsequent NO generation to regulate vascular function (Kobayashi et al., 2005). Impairment in PI3K signaling pathway has been demonstrated in aortas of diabetic mice (Kobayashi et al., 2004). The activated PI3K-Akt signaling pathway can also improve insulin sensitivity and protect the vascular endothelium (Feng et al., 2018).

Activation of P13K/Akt/eNos pathway, such as by components of green tea, has been reported to have beneficial effects on diabetes-induced vascular dysfunction (Bhardwaj et al., 2014; Zhang and Zhang, 2020). For example, (-)-Epigallocatechin-3-gallate (EGCG), a highly effective component in green tea that has anti-inflammatory as well as antioxidant and free radical scavenging properties, was able to inhibit eNOS uncoupling and prevent hyperglycemia-induced endothelial dysfunction and apoptosis by activating the PI3K/AKT/eNOS pathway (Zhang and Zhang, 2020). Similarly, in a rat model of T1DM, chronic administration of catechin, another key component of green tea, markedly attenuated diabetes-induced vascular dysfunction and vascular oxidative stress via activation of endothelial PI3K signaling and the subsequent downstream eNOS signaling system that generates NO (Bhardwaj et al., 2014). Although PI3K-eNOS signaling can be a downstream consequence of EGFR activation, the relationship between EGFR activation in diabetic vasculature and administration of green tea components was not investigated in these studies. Nonetheless, these studies suggest that PI3K signalling is beneficial in maintaining vascular function as its attenuation is associated with diabetes induced vascular complications.

In contrast to these studies, in a rat model of TIDM, chronic selective inhibition of PI3K with LY294002 significantly attenuated the development of diabetes-induced abnormal vascular reactivity in the carotid artery (Yousif et al., 2006) implying that that PI3K pathway might also mediate vascular dysfunction. The reasons for this discrepancy are not clear but several reports have now confirmed AKT, and by implication PI3K to which it often coupled, as a downstream player in ErbB receptor-dependent vascular dysfunction in models of diabetes (Benter et al., 2015; Kasan et al., 2015b; Amin et al., 2011). For example, in the diabetic mesenteric vascular bed, hyperglycemia-induced upregulation of Akt signaling was prevented by lapatinib, a dual inhibitor of EGFR and ErbB2 (Benter et al., 2015). With the caveat that AKT signaling does not always have to be coupled to PI3K, these studies are suggestive of a possible detrimental role of PI3K/AKT pathway in diabetes-induced vascular dysfunction. Supportive of this notion, is the finding that ANG II, that is known to be elevated in diabetes, increases aortic contractile responses via PI3-kinase pathway in a rat model of diabetes with systemic hyperinsulinemia (Kobayashi et al., 2006). Collectively, the data on the role of PI3K in mediating vascular dysfunction appears contradictory and may be indicative of a dual role for this molecule whereby depending on the context it too may behave as a good guy or a bad guy, akin to the role of EGFR/ErbB receptors in cardiovascular complications as discussed herein.

### Interplay of Epidermal Growth Factor Receptor and Forkhead Transcription Factors in Diabetes-Induced Vascular Dysfunction

FOXO3 is a pro-apoptotic protein that belongs to the forkhead family of transcriptional regulators which are critical mediators of oxidative stress (Ponugoti, et al., 2012). FOXO activity, amongst others, is regulated via phosphorylation, whereby increased phosphorylation renders it inactive via mechanisms including nuclear exclusion, polyubiquitination, and degradation (Oellerich and Potente, 2012). ROS/oxidative stress can induce FOXO3 phosphorylation which results in its release from the 14–3-3 binding protein, and subsequent translocation to the nucleus where it modulates target gene expression (Ponugoti, et al., 2012). The role of FOXO3 as a downstream effector of EGFR/ErbB2 receptors was studied in the mesenteric vascular bed of T1DM rats (Benter et al., 2015). Diabetes-induced vascular dysfunction arising from enhanced EGFR/ErbB2 signaling also led to downstream activation of FOXO3 (as evidenced by reduced phosphorylation at Ser253) through an AKT-independent manner that ultimately leads to apoptosis and vascular dysfunction (Benter et al., 2015). Hyperglycemia-mediated decrease in FOXO3A phosphorylation and increased total FOXO3A levels could be reversed upon lapatinib treatment (a dual inhibitor of EGFR and ErbB2 receptors) (Benter et al., 2015). Thus, FOXO-mediated pro-apoptotic signaling is likely an important downstream contributor to EGFR/ErbB receptor-mediated vascular dysfunction in diabetes.

### The Link Between Epidermal Growth Factor Receptor and NF-kB Transcription Factor in Diabetes-Induced Vascular Dysfunction

The Nuclear Factor Kappa B (NF-kB) transcription factor is a key player in the development of diabetes-induced vascular complications through regulation of many cellular processes such as inflammation, cell growth, proliferation, and apoptosis (Suryavanshi and Kulkarni., 2017; Taniguchi and Karin, 2018). In unstimulated cells, inactive NF-kB is bound in the cytoplasm to inhibitory proteins in the inhibitor of kB (IkB) family. Activation of NF-kB occurs via signal transduction pathways that stimulate the IkB kinase (IKK) complex resulting in a series of reactions that ultimately cause degradation of IkB and releasing of active NF-kB. Aberrant NF-kB activity has been implicated in many diseases including diabetes and its complications. Chronic hyperglycemia is known to activate NF-kB that triggers expression of pro-inflammatory cytokines and pro-apoptotic genes. It can also induce calcification of endothelial cells leading to endothelial and vascular dysfunction (Suryavanshi and Kulkarni., 2017). In the vasculature of T1DM rats, diabetes leads to the enhanced phosphorylation of IkB-α (Benter et al., 2015), a component of IKK complex and a universal marker of NF-kB activation. Data from this study illustrated that the increased ErbB2-EGFR hetero-dimerization in the vasculature leads to elevated NF-kB activation, which eventually induced apoptosis and vascular dysfunction (Benter et al., 2015). This was evidenced by *in vivo* and *ex vivo* lapatinib administration which attenuated the increased phosphorylation of IkB-α in VSMCs grown under high glucose conditions (Benter et al., 2015). Furthermore, administration of BAY 11–7,082, an NF-kB antagonist, corrected the altered vascular responsiveness to norephinephrine in the diabetic mesenteric vascular bed and hyperglycemia-induced apoptosis in VSMCs (Benter et al., 2015). Thus, it was determined from these results that NF-kB is downstream of EGFR-ErbB2 signaling in T1DM (Benter et al., 2015). These findings were also confirmed in coronary arteries of mice bearing T2DM (Kassan et al., 2015b). Intriguingly, it was determined that eNOS signaling was coupled with NF-kB activation in the diabetic mesenteric vasculature, where eNOS was shown to be upstream of NF-kB in mediating vascular dysfunction associated with diabetes (Benter et al., 2015).

### The Link Between Epidermal Growth Factor Receptor & Nicotinamide Adenine Dinucleotide Phosphate Oxidase in Diabetes-Induced Vascular Dysfunction

Nicotinamide adenine dinucleotide phosphate (NADPH) oxidase (NOX) enzyme complex comprises of several sub-units, namely, p22phox, NOX2; p47phox; p67phox; and p40phox; also known as NOX1, NOX2, NOX3, NOX4, and NOX5 respectively (Volpe et al., 2018). It is well established that the NOX family are major producers of reactive oxygen species that mediate cardiovascular complications associated with diabetes (Volpe et al., 2018; Iacobini et al., 2021). Several studies have now shown that EGFR inhibition leads to a reduction in NOX activity in models of diabetes-induced. cardiovascular complications (Belmadani et al., 2008; Choi et al., 2012; Galan et al., 2012; Kassan et al., 2015a)- implying that EGFR/ErbB receptor activation and subsequent NOX-mediated ROS/oxidative stress are critical in mediating vascular dysfunction associated with diabetes. In the mesenteric artery, mRNA levels of Nox2 and Nox4 and NADPH oxidase activity were upregulated in a mouse model of T1DM and reduced upon EGFR inhibition (Galan et al., 2012). Similarly, in an experimental model of TD2M, selective EGFR inhibition improved vascular function and reduced p22phox-NADPH expression (Kassan et al., 2015b). Collectively these studies provide a direct link between EGFR and NADPH oxidase in mediating oxidative stress that leads to diabetes-induced vascular dysfunction.

### The Interplay of Epidermal Growth Factor Receptor and Angiotensin 1-7, a Member of the Renin-Angiotensin-Aldosterone System, in Diabetes-Induced Vascular Dysfunction

The renin-angiotensin-aldosterone system (RAAS) is a key regulator of homeostasis within the cardiovascular system (Ferrario et al., 2010; Akhtar et al., 2020a; Paz Ocaranza 2020). Its main peptide, Angiotensin II (Ang II), is known to be detrimental in the diabetic vasculature where it mediates oxidative stress, pro-inflammatory signaling and vasoconstriction. Enhanced signaling via this octapeptide in the diabetic vasculature involves transactivation and cross-talk with EGFR/ErbB/HER family of receptors (Akhtar et al., 2012a; Akhtar et al., 2015; Akhtar et al., 2016; Sur and Agrawal, 2014).

Ang II-mediated detrimental effects on the vasculature are exerted through the classical arm of the RAAS constituting angiotensin converting enzyme (ACE)/Ang II/Angiotensin II type 1 (AT1) receptors. The other arm of the RAAS that counter-regulates or opposes the actions of Ang II involves the heptapeptide, Angiotensin-(1–7) and comprises ACE2/Ang-(1–7)/Mas receptor (for recent review see Akhtar et al., 2020a). Ang-(1–7) can be derived from the catalytic metabolism of Ang II via the actions of angiotensin converting enzyme 2 (ACE2) (Ferrario et al., 2010). Ang-(1–7) induces vasodilation and attenuates Ang II-induced vasoconstriction in animal models (Benter et al., 1993). In humans, Ang-(1–7) also improves insulin-stimulated endothelium-dependent vasodilation and attenuates endothelin-1–dependent vasoconstrictor tone in obese patients (Schinzari et al., 2018). In endothelial cells Ang-(1–7) increases production of nitric oxide and prostacyclin and in vascular smooth muscle cells it inhibits pro-contractile and pro-inflammatory signalling (Benter et al., 1993; Benter et al., 1995a; Ferrario et al., 2010). It is known that Ang-(1–7) exhibits antihypertensive, antithrombotic and antiproliferative properties and can correct abnormal vascular reactivity including that observed in diabetes (Benter et al., 1995a; Benter et al., 1995b; Benter et al., 2006; Benter et al., 2007; Benter et al., 2008; Benter et al., 2011; Chappell et al., 2014; Dhaunsi et al., 2010; Patel et al., 2014; Yousif et al., 2012; Yousif et al., 2014). In animal models of diabetes, Ang-1-7 can attenuate NADPH oxidase and NF-kB activity and prevent vascular dysfunction without markedly correcting hyperglycemia (Benter et al., 2007; Benter et al., 2008; Al-Maghrebi et al., 2009; Yousif et al., 2014). Although the precise mechanistic details through which Ang-(1–7) exerts its beneficial actions are not yet fully elucidated, it appears that Ang-(1–7) prevents diabetes-induced vascular dysfunction, at least in part, via inhibiting Ang II-mediated transactivation of EGFR/ErbB family of receptor tyrosine kinases (Akhtar et al., 2012a; Akhtar et al., 2015). In addition to inhibiting EGFR phosphorylation at multiple tyrosine sites in the vasculature of diabetic animals, Ang-(1–7) inhibited ErbB2 phosphorylation at tyrosine residues Y1221/22, Y1248, Y877, with modulation of associated downstream signaling pathways involving ERK1/2, p38 MAPK, ROCK, eNOS, and lkB-α in the diabetic mesenteric vascular bed (Akhtar et al., 2015). Furthermore, high glucose- or diabetes-induced ErbB3, and Erbb4 transactivation could be ameliorated by Ang-(1–7) treatment thereby suggesting that Ang-(1–7) acts as a pan-inhibitor of the EGFR/ErbB/HER family of receptor tyrosine kinases (Akhtar et al., 2015). More recently other pan-ErbB inhibitors have been shown to be beneficial in reversing or preventing diabetes-induced vascular dysfunction (Akhtar et al., 2019; Akhtar et al., 2020b) and as such pan-ErbB inhibition might prove to be a useful strategy in the treatment of diabetes induced vascular complications.

As to the mechanism of diabetes-induced transactivation of ErbB receptors, high glucose-mediated ErbB2 transactivation occurred via a Src-dependent mechanism in VSMCs as evidenced by increased phosphorylation of Src at Y416. Ang-(1–7) acting via its MAS receptor blocked the phosphorylation of Src at this site-an upstream effector of ErbB2 transactivation (Akhtar et al., 2015). Thus, it was proposed that Ang-(1–7) inhibited diabetes or hyperglycemia induced transactivation of ErbB receptors in the diabetic vasculature by acting as an inhibitor of Src (Akhtar et al., 2012a; Akhtar et al., 2015). Subsequent studies in cardiac fibroblasts (Tao et al., 2014) and in activated macrophages (Souza and Costa-Neto, 2012) further supported the notion that Ang-(1–7) can inhibit Src phosphorylation. Recent studies in other disease models suggest that inhibition of Src-dependent EGFR transactivation by Ang-(1–7) may be a general mechanism of action of this heptapeptide (Kilarkaje et al., 2013; Yousif et al., 2014; El-Hashim et al., 2019) and may represent an important mechanism by which it may exert its beneficial effects in diabetes-induced vascular complications.

It is noteworthy that, despite its inhibitory effects on EGFR and other ErbB receptors (Akhtar et al., 2012b; Akhtar et al., 2015), Ang-(1–7) exerts considerable cardioprotective effects in models of diabetes (Dhaunsi et al., 2010; Yousif et al., 2012; Benter et al., 2014; Abwainy et al., 2016). Thus, the precise mechanisms underlying this phenomenon, especially as to how Ang-(1–7) interplays with the dual role of EGFR/ERbB signaling in the CVS, remain to be elucidated.

### The Arachidonic Acid Metabolite, 20-HETE and Its Interplay With Epidermal Growth Factor Receptor in Diabetes-Induced Vascular Dysfunction

The arachidonic acid metabolite, 20-HETE (20-hydroxy-5, 8, 11, 14-eicosatetraenoic acid), plays an important role in controlling cardiovascular function (Rocic and Schwartzman, 2018; Yousif et al., 2009a; Yousif et al., 2009b). In blood vessels, it acts as a potent vasoconstrictor, regulates myogenic tone and also potentiates the vasoconstrictor response to peptides like angiotensin II and endothelin (Roman 2002; Fan et al., 2016). 20-HETE induces endothelial dysfunction and adverse vascular remodeling that leads to increased blood pressure (Fan et al., 2016). Its actions are most likely exerted through its newly discovered receptor, GPR75, an orphan G-protein (Gα_q/11_) coupled receptor (Garcia et al., 2017).

20-HETE is produced from arachidonic acid metabolism, not via the classical cyclooxygenase (COX) or lipoxygenase (LOX) pathways, but by a third pathway involving cytochrome P450-mediated ω-hydroxylation via CYP4A and CYP4F enzyme sub-families (Fan et al., 2016; Fan et al., 2015; Roman, 2002). Its presence has been reported in the microvasculature of the brain, lungs, kidneys, and the peripheral blood vessels (Miyata and Roman, 2005). There is accumulating evidence that CYP4A/4F and 20-HETE pathway play a major role in vascular dysfunction and changes in 20-HETE levels is implicated in many diseases like diabetes and hypertension (Fan et al., 2016; Miyata and Roman, 2005; Rocic and Schwartzman, 2018; Yousif et al., 2009a; Yousif et al., 2009b). For example, 20-HETE contributed to elevated vascular reactivity in renal and mesenteric vasculature of streptozotocin-induced diabetic mice (Yousif et al., 2009a). Moreover, 20-HETE stimulates mitogenic and angiogenic responses (*in vivo* and *in vitro*) to various growth factors such as EGF (Miyata and Roman, 2005). This was demonstrated in a study whereby inhibition of 20-HETE by HET0016 (an inhibitor of 20-HETE synthesis) almost completely abolished the angiogenic responses of EGF and other growth factors (Chen et al., 2005). Also, 20-HETE has been implicated as a key regulator of NO production and function. For instance, 20-HETE leads to eNOS uncoupling and impairs acetylcholine-induced relaxation through tyrosine kinase-, MAPK/ERK-, and IKK- dependent mechanisms that involve HSP90-eNOS dissociation and HSP90-IKKβ association (Cheng et al., 2010) leading to endothelial dysfunction. Moreover, 20-HETE stimulates NF-kB and increases levels of proinflammatory cytokines causing endothelial activation. This is partly mediated via MAPK-ERK1/2 signalling (Ishizuka et al., 2008).

Further studies on the mechanism by which 20-HETE exerts its actions in the vasculature (Chen, et al., 2012; Guo et al., 2007; Guo et al., 2009) showed that 20-HETE stimulates endothelial progenitor cell proliferation, migration and tube formation as well as enhancing release of proangiogenic factors such as VEGF. Importantly, these studies revealed the interplay with EGFR/ErbB receptors in that these vascular effects of 20-HETE were mediated through Src and EGFR with downstream activation of NADPH oxidases/reactive oxygen species (ROS) system, eNOS uncoupling and stimulation of MAPK and PI3K/Akt pathways (Chen, et al., 2012; Guo et al., 2007; Guo et al., 2009).

In VSMC, it was shown that norephinephrine (NE), Ang II, and EGF can activate the Ras/MAPK pathway through generation of 20-HETE (Muthalif et al., 1988). Since ACE/Ang II/AT1 receptor pathway is known to lead to transactivation of ErbBs in VSMC (Akhtar et al., 2015; Akhtar et al., 2016), the fact that 20-HETE is a potent activator of endothelial ACE expression and activity might represent another point of interplay between EGFR and 20-HETE signaling. Indeed, 20-HETE induces ACE expression through increased NF-κB binding to the ACE promoter (Cheng et al., 2012; Garcia et al., 2016). Endothelial ACE transcription is in turn mediated by EGFR via a MAPK/IkappaB kinase signaling cascade that ultimately results in increased ACE activity (Garcia et al., 2016). Furthermore, in parallel enhanced synthesis of 20-HETE *in vivo* leads to increased ACE expression in the vasculature (Garcia et al., 2015; Garcia et al., 2016; Garcia et al., 2017; Garcia and Schwartzman, 2017) as well as increased serum Ang II levels (Sodhi et al., 2010). Blocking 20-HETE synthesis prevented these changes, implicating 20-HETE in the regulation of ACE expression and AngII levels *in vivo* that indirectly could have an impact on ACE/AngII/AT1 receptor mediated transactivation of ErbBs. Thus, EGFR is likely involved in mediating the actions of 20-HETE in stimulating ACE expression and Ang II levels as well being involved in the downstream actions of ACE/AngII/AT1 receptor signaling possibly via Src phosphorylation. Collectively the above studies suggest that EGFR/ErbB receptor transactivation may represent an important signaling convergence point or “hub” by which many cardiovascular risk factors promote diabetes-induced vascular dysfunction and remodeling.

## Perspectives and Concluding Remarks

Signaling via EGFR/ErbB family of receptor tyrosine kinases appears to be a central hub or relay in mediating vital functions in the normal development and physiology of the cardiovascular system as well as in the development of cardiovascular complications and pathologies. All four members, ErbB1-4, appear to be expressed both in the normal and diabetic heart as well as normal and diabetic vasculature where EGFR/ErbB receptors appear to have a dual (or “good guy” v “bad guy”; “Jekyll and Hyde” or “Janus-faced”) role. EGFR/ErbB receptors generally have beneficial “good-guy” actions in normal physiological function of the cardiovascular system and in mediating key “salvage” or recovery pathways following cardiac ischemia-reperfusion injury. However, several studies highlighted in this review confirm also the detrimental “bad-guy” effect of upregulated EGFR/ErbB signaling in the pathogenesis of diabetic-induced cardiac pathologies (Galan et al., 2012; Liang et al., 2015). Further highlighting the dual role of EGFR/ErbB receptors, these findings apparently contradict those showing protective effects of EGFR/ErbBs following ischemia/reperfusion injury in diabetic animals (Akhtar et al., 2012a). In an attempt to reconcile this contradiction, it has been postulated that EGFR/ErbB activation could be protective in the context of acute injury such as acute ischemia/reperfusion injury, but detrimental in chronic pathologies where persistent upregulation of EGFR/ErbB receptor signaling is observed such as diabetes-induced cardiac fibrosis and remodeling (Xu and Cai, 2015).

**FIGURE 2:**
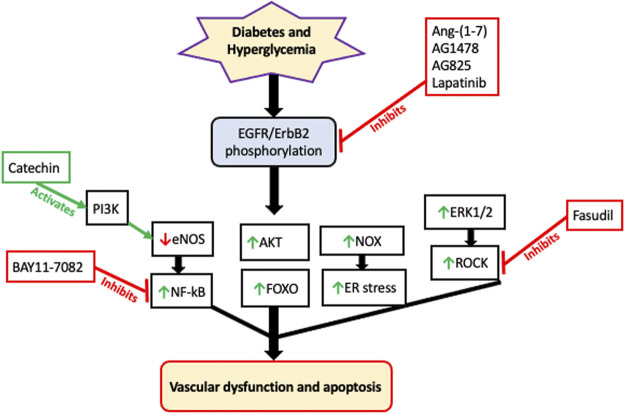
A schematic of the likely signaling effectors of EGFR/ErbB receptors in mediating diabetes-induced vascular dysfunction. Although there is now evidence that all four ErbB receptors may be activated by hyperglycemia, the majority of the data suggests that diabetes induced vascular dysfucntion proceeds via phosphorylation and subsequent hetero-dimerization EGFR and ErbB2 receptors. This is turn begins a cascade of downstream signaling pathways including attenuation of eNOS that results in decreased NO production. Activation of P13K, such as by green tea components like catechin, can rescure eNOS and reduction in NO. eNOS appears to be upstream of NF-kB activation. Additionally, ERK1/2 activation leads to downstream ROCK signaling–a key driver of pro-contractile changes leading to vascular dysfunction. Interestingly, activation of AKT and FOXO occur independently of each other implying that uncoupling of AKT-FOXO axis is a key component in developing diabetes-induced vascular complications. There is also evidence that EGFR/ErbB2 signaling via NADPH-oxidase (NOX) leads to increased ROS/oxidative and ER stress in the diabetic cardiovascular system. The precise cross-talk and interplay between these and other pathways remains to be fully elucidated. Furthermore, since multiple gene expression changes are known to corrected by EGFR inhibition in the diabetic vasculature, it is very likely that the signaling pathways discussed here will act in concert with multiple other, as yet unconfirmed, signaling cascades that are downstream of EGFR/ErbB receptors to eventually lead to diabetes-induced vascular cell apoptosis and dysfunction. Some of the reported pharmacologic interventions used in the analyses of these pathways are also shown; red dead-end arrow indicates inhibition; green arrow indicates activation (refer to the main text for more details and references).

Similarly, upregulation of all four ErbBs has been implicated in the development of diabetes-induced vascular dysfunction through mechanisms involving multiple downstream effectors such as ERK1/2, ROCKs, p38 MAPK, FOXO, AKT, eNOS, and NF-kB [see also Figure 2] (Akhtar et al., 2015; Akhtar et al., 2020a). Both in the vasculature and in the heart, much of the evidence suggests that EGFR/ErbB receptors might function as key relays for the actions of the RAAS especially in mediating the pathological actions of its detrimental arm comprising the ACE/Ang II/AT1 receptor signaling cascade-that is known to lead to transactivation of EGFR/ErbBs with subsequent development of cardiovascular pathologies. Similarly, EGFR/ErbB receptors appear to be a key target for the actions of the beneficial counter-regulatory, ACE2/Ang-(1–7)/Mas receptor, pathway or “arm” of the RAAS. For example, the vasculo-protective effects of Ang-(1–7)/Mas receptor appear to be mediated, at least in part, via blockade of a Src-dependent EGFR/ErbB receptor phosphorylation and attenuation of downstream effectors (Akhtar et al., 2012a; Akhtar et al., 2015).

Interestingly, EGFR/ErbB receptor signaling also appears important in mediating the vascular effects of the arachidonic acid metabolite, 20-HETE-a key regulator of cardiovascular function that also exhibits significant cross-talk with RAAS. EGFR/ErbB receptors are likely involved in mediating the actions of 20-HETE especially those leading to enhancing ACE expression and serum Ang II levels. Further, EGFR/ErbB receptors are also likely be involved in the downstream actions of ACE/AngII/AT1 receptor signaling such as in the Src-dependent transactivation of EGFR/ErbB receptor family members that ultimately leads to diabetes-induced vascular complications. Therefore, EGFR/ErbBs might represent a key point of convergence for actions of the RAAS and other molecules that are involved in cross-talk with RAAS such as 20-HETE. Thus, targeting ErbB receptor might represent a novel strategy for the treatment of cardiovascular complications.

EGFR inhibition is reported to have several benefits in diabetes including normalization of cardiovascular function, reduction of blood pressure, lowering of blood glucose levels and reversing insulin resistance (Hao et al., 2004; Nagareddy et al., 2010; Akhtar et al., 2013; Kassan et al., 2015a; Akhtar et al., 2015; Benter et al., 2015; Fountas et al., 2015). Inhibitors of EGFR/ErbB receptors (e.g., lapatinib) are already clinically available for the treatment of several cancers but could also be repurposed for treatment of diabetes-induced cardiovascular complications (Akhtar et al., 2015). In contrast, inducers or activators of EGFR/ErbB receptors (e.g., NRG or EGF) might be useful in recovering hearts from ischemia-reperfusion injury especially in diabetes where cardiac function is already compromised. Interestingly, in this regard it was found that the beneficial actions of RAAS inhibitors such as the angiotensin receptor blockers (e.g., losartan), that are clinically approved for use in myocardial infarction/ischemic heart disease, might not be optimal and cardiac function recovery could be improved by co-administering ligands or activators of EGFR/ErbB signaling (Akhtar et al., 2012b). Consistent with losartan’s known inhibition of Angiotensin II-mediated transactivation of EGFR (e.g., Akhtar et al., 2016), reduced EGFR phosphorylation was observed in losartan-treated diabetic hearts following ischemia-reperfusion injury compared to untreated diabetic hearts. Co-treatment of losartan with EGF, a ligand for EGFR, prevented the inhibitory effects of losartan on EGFR transactivation with a parallel improvement in cardiac function recovery greater than either agent alone. These findings may have important clinical implications as they suggest that rescuing the EGFR inhibitory effect of AT1 receptor antagonists by activators of the EGFR/ErbB family of receptor tyrosine kinases may represent a novel clinical approach to improve protection against end-organ damage in diabetic hearts. However, future treatments targeting EGFR/ErbBs will need to balance their beneficial versus the detrimental effects in a given cardiovascular pathology as well as the differential expression of EGFR/ErbB receptors and their ligands in different tissues. A greater understanding of the multi-faceted roles of EGFR/ErbB/HER family of tyrosine kinases, their interplay with other key modulators of cardiovascular function, and the development of smart or intelligent drug delivery systems (e.g., Mitchel et al., 2021) that can spatially and temporally control delivery of EGFR/ErbB/HER receptor modulators for a specific application, could facilitate the development of novel therapeutic strategies/formulations for treating diabetes-induced cardiovascular complications. Indeed, engineered nanoparticles, that could potentially encapsulate and site-specifically deliver EGFR/ErbB receptor modulators for a specific purpose, are now in advanced stages of development (Mitchell et al., 2021). By controlling nanoparticle surface and nanomaterial properties, their size, shape and architecture, as well as through careful selection of cell or tissue-specific targeting moieties and built-in responsiveness to environmental cues such as hyperglycemia, nanoparticle delivery systems can now be produced with intelligent designs to tailor the delivery of EGFR/ErbB receptor drugs to a specific application or condition as new-age precision medicines. Such approaches may have broader application beyond diabetes-induced cardiovascular dysfunction as dysregulated EGFR/ErbB/HER signaling also leads to diabetes-induced retinal and corneal complications (Akhtar et al., 2009; Ju et al., 2019), diabetic foot ulcers (Qi et al., 2018; Yang et al., 2020) and renal pathologies (Harskamp et al., 2016; Rayego-Mateos et al., 2018; Sheng et al., 2021) implying that aberrant signalling via EGFR/ErbB/HER receptors and their ligands might represent a common underlying mechanism in the development of diabetes complications.
